# False positives complicate ancient pathogen identifications using high-throughput shotgun sequencing

**DOI:** 10.1186/1756-0500-7-111

**Published:** 2014-02-25

**Authors:** Michael G Campana, Nelly Robles García, Frank J Rühli, Noreen Tuross

**Affiliations:** 1 Department of Human Evolutionary Biology, Harvard University, Peabody Museum, 11 Divinity Avenue, Cambridge, MA 02138, USA; 2 Centre for Evolutionary Medicine, Anatomy Institute, University of Zurich, 190 Winterthurerstrasse, Zurich 8057, Switzerland; 3 Instituto Nacional de Antropología e Historia, Mexico City, Mexico

**Keywords:** True single molecule sequencing, High-throughput sequencing, Pathogen, Ancient DNA, False positive

## Abstract

**Background:**

Identification of historic pathogens is challenging since false positives and negatives are a serious risk. Environmental non-pathogenic contaminants are ubiquitous. Furthermore, public genetic databases contain limited information regarding these species. High-throughput sequencing may help reliably detect and identify historic pathogens.

**Results:**

We shotgun-sequenced 8 16th-century Mixtec individuals from the site of Teposcolula Yucundaa (Oaxaca, Mexico) who are reported to have died from the *huey cocoliztli* (‘Great Pestilence’ in Nahautl), an unknown disease that decimated native Mexican populations during the Spanish colonial period, in order to identify the pathogen. Comparison of these sequences with those deriving from the surrounding soil and from 4 precontact individuals from the site found a wide variety of contaminant organisms that confounded analyses. Without the comparative sequence data from the precontact individuals and soil, false positives for *Yersinia pestis* and rickettsiosis could have been reported.

**Conclusions:**

False positives and negatives remain problematic in ancient DNA analyses despite the application of high-throughput sequencing. Our results suggest that several studies claiming the discovery of ancient pathogens may need further verification. Additionally, true single molecule sequencing’s short read lengths, inability to sequence through DNA lesions, and limited ancient-DNA-specific technical development hinder its application to palaeopathology.

## Background

Diseases have ravaged human populations throughout history. The pathogens responsible for many historic epidemics remain either unknown or speculative (e.g. the Plague of Athens [1,2]). Identification of these historic pathogens is critical to understanding disease evolution, which in turn has direct impacts on the development of effective medical treatments for these conditions. The discovery that ancient pathogen-diagnostic biomolecules may survive in archaeological bones and the development of high-throughput DNA sequencing technologies have opened new possibilities for determining past disease [3-14].

Most biomolecular palaeopathological studies have been based on the polymerase chain reaction (PCR) (e.g. [3-7]). Supplementary methods include lipid and immunological assays [6-12]. Due to the limitations of molecular preservation and detection, negative assays do not constitute evidence of absence of any infection [15-17]. False positives are a serious concern since bacteria, viruses and fungi are ubiquitous and the specificity of molecular tests may not be able to distinguish between target disease agents and their closely related relatives [18-20]. Many PCR-based palaeopathological reports are contentious due to insufficient anti-contamination procedures or lack of experimental and analytical rigor [21-23].

Furthermore, PCR-based methods are limited in their ability to identify unknown pathogens since they require candidate disease agents of known sequence in advance. Although there are a few studies that attempt such *de novo* identification (e.g. [5,24]), the vast majority of investigations have been restricted to targeted, likely diseases. While DNA capture methods, including in-solution [e.g. [13] and array-hybridization [e.g. [14] capture, have become the gold standard for palaeopathogenomics, no such technology is yet available for palaeopathogen diagnosis. In theory, it is possible to identify unknown diseases by high-throughput DNA sequencing of known afflicted individuals and comparing these pools with suitable controls (such as the surrounding soil and uninfected archaeologically related individuals). Organisms found only in the infected population would thus be candidates for the responsible pathogens. Nevertheless, since the majority of sequences in most ancient DNA samples are derived from the environment (particularly soil bacteria), false positives could remain problematic in high-throughput analyses [23,25]. Moreover, the public genetic databases are both biased towards pathogenic cellular organisms and deficient in virus sequences, thereby increasing the false positive rate for cellular pathogens while simultaneously raising the false negative rate for viral infections in *de novo* identifications.

Here we show that false positives are a significant error source in palaeopathological analyses using high-throughput sequencing. Using two platforms (the Helicos BioSciences HeliScope and the Illumina HiSeq 2500), we attempted to identify *de novo* the pathogen responsible for the *huey cocoliztli* (‘Great Pestilence’ in Nahautl), a disease that ravaged the native Mexican population during the Spanish colonial period [26].

## Methods

### DNA extraction

In order to identify the *huey cocoliztli* pathogen, we analyzed the site of Teposcolula Yucundaa (Oaxaca, Mexico), a large Mixtec city at the time of Spanish colonization [27]. The site was abandoned in 1552 following a major outbreak of the *huey cocoliztli* in the 1540 s. Excavations at Teposcolula Yucundaa conducted between 2004 and 2006 revealed two Mixtec cemeteries: a colonial period graveyard (the Grand Plaza) and a smaller precolonial one (the Churchyard) [27]. Warinner and colleagues [27] identified the Grand Plaza graveyard as a plague pit corresponding to the 1540 s pandemic of the *huey cocoliztli*. The precontact Churchyard population is assumed to have been uninfected with the *huey cocoliztli* since the native Mexican populations were unfamiliar with the disease at the time of its first appearance during the colonial period [28]. DNA preservation at Teposcolula Yucundaa is exceptional, probably due to the high-altitude, cool environment and relatively recent date [27].

Twelve femoral cortical bone specimens (each representing a unique individual; Table 1) were collected in the field and ground to a fine power (described in [29]). DNA extractions were performed in a dedicated ancient DNA laboratory in the Department of Human Evolutionary Biology, Harvard University according to standard anti-contamination protocols [30]. Approximately 1 g of bone powder per individual was decalcified in 0.5 M EDTA, pH 8.0. Raw DNA extract was passed through a vacuum filter to remove residual protein and powder and then concentrated to ~500 μl via ultrafiltration using Vivaspin® 20’s (Vivaproducts) with a 10 kDa molecular weight cut-off. Concentrated extracts were treated with proteinase K. Protein-digestion completion was verified using a Qubit® 2.0 fluorometer (Life Technologies). Digested extracts were then purified using QIAquick PCR Purification Kits (Qiagen). Bulk DNA extracts consisting of 4 individuals per bulk were constructed for the Grand Plaza (individuals TP02, TP10, TP15, TP26) and Churchyard (individuals TP32, TP42, TP45, TP48) populations. Bulking the samples increased the likelihood of detecting the pathogen since only a fraction of the infected individuals are expected to have endogenous disease DNA preserved [e.g. [13,14].

**Table 1:** Teposcolula Yucundaa individuals analyzed here

**Individual**	**Graveyard**	**Sex**	**Age**
TP02	Grand Plaza	Female	25 ± 1
TP04	Grand Plaza	Female	26 ± 2
TP09	Grand Plaza	Male	20 ± 2
TP10	Grand Plaza	Male	20-21
TP15	Grand Plaza	Indeterminate	Adolescent
TP18	Grand Plaza	Female	35 ± 4
TP26	Grand Plaza	Female	40 ± 4
TP32	Churchyard	Female	19 ± 1
TP37	Grand Plaza	Female	Young Adult
TP42	Churchyard	Indeterminate	5
TP45	Churchyard	Female	36
TP48	Churchyard	Female	32 ± 2

Sample details are from [27,29].

Three samples of soil from the Grand Plaza and the Churchyard cemeteries were also collected. Soil was collected from the burial contexts (within a few centimeters of the skeletons) by trowelling samples directly into collection bags while wearing gloves to limit DNA contamination. DNA was extracted from 5–8 g of soil per sample using the PowerMax® Soil DNA Isolation Kit (Mo-Bio Laboratories). Purified extracts from each burial were then combined into a bulk sample.

Finally, subsamples of all three bulk extracts (Churchyard, Grand Plaza and soil) were sheared to 150 bp average length using a S220 Focused-ultrasonicator (Covaris, Inc.) for subsequent true single molecule sequencing (tSMS). Although shearing DNA extracts is typically denigrated in high-throughput ancient DNA analyses (e.g. [31]), the sequences generated by tSMS are shorter (typically <40 bp) than the endogenous DNA molecules known to be present in the Teposcolula Yucundaa individuals by PCR (~100 bp) [27] and Bioanalyzer assays (see below). Since tSMS only generates one sequence per molecule and has limited ability to sequence through DNA lesions, shearing might make more of the endogenous DNA sequenceable (but at the cost of a possible increase in microbial contamination) by producing multiple 3′-termini per original molecule. Additionally, six extracts representing single individuals (TP04, TP09, TP10, TP18, TP37, TP48) and a subsample of the soil bulk extract were sheared to 150–200 bp average length for subsequent Illumina library construction using the automated Apollo 324 (IntegenX) platform.

DNA concentrations for all individual extracts, bulks and sheared bulks were calculated using a high-specificity DNA kit on a Qubit® fluorometer. Length distributions of DNA molecules in these samples were calculated using a high-specificity DNA chip on an Agilent 2100 Bioanalyzer (Agilent Biotechnologies).

### Helicos HeliScope sequencing

A total of 12 channels of tSMS was performed on a Helicos HeliScope sequencer at Helicos BioSciences (Cambridge, MA). Eight μl per channel of each bulk and sheared bulk were prepared for tSMS according to the standard protocol for ancient samples [32]. Two channels of each bulk and one channel of each sheared bulk were sequenced. Additionally, samples of the Churchyard and Grand Plaza bulk extracts were treated with Antarctic Phosphatase (New England Biolabs), diluted to the equivalent concentration of their untreated counterparts and prepared for HeliScope sequencing as described in [33]. Phosphatase-treatment may increase HeliScope sequence yield [33]. Two channels of each phosphatase-treated bulk were sequenced. The bulks and phosphatase-treated bulks were sequenced on one chip, while the soil and sheared bulks were sequenced on another. For the unsheared Churchyard and Grand Plaza bulks (both phosphatase-treated and untreated samples), 23–31 million quality-controlled sequences were generated per channel (Table 2). There were technical issues with the sheared and soil samples (see below), so only between 4,000 and 840,000 reads per channel were obtained for these four channels (Table 2).

**Table 2:** Sequencing statistics for the studied samples

**Platform**	**Sample Description**	**No. Filtered Sequences**	**Molecular Length (bp)**	**Total Sequenced (bp)**
HeliScope	Grand Plaza Bulk 1	31,183,210	34.1 ± 7.1	1,063,972,634
	Grand Plaza Bulk 2	30,773,387	34.4 ± 7.2	1,058,435,915
	Grand Plaza Phos. 1	26,269,497	34.4 ± 7.3	904,192,902
	Grand Plaza Phos. 2	23,061,503	35.0 ± 7.4	806,304,129
	Churchyard Bulk 1	28,960,308	33.9 ± 7.0	981,718,381
	Churchyard Bulk 2	27,035,110	33.8 ± 7.0	914,002,286
	Churchyard Phos. 1	28,423,683	34.5 ± 7.3	981,600,068
	Churchyard Phos. 2	29,660,255	33.8 ± 7.0	1,003,204,069
	Grand Plaza Sheared	4,088	27.1 ± 3.4	110,619
	Churchyard Sheared	5,309	27.2 ± 3.5	144,140
	Soil Bulk	840,915	33.4 ± 7.2	28,082,034
	Soil Sheared	123,211	31.4 ± 6.4	3,869,834
HiSeq 2500	TP04	9,643,548	219.5 ± 43.9	2,117,000,413
	TP09	6,640,789	236.2 ± 39.2	1,568,449,339
	TP10	5,368,267	223.7 ± 40.3	1,201,119,486
	TP18	7,693,734	215.5 ± 39.1	1,658,247,428
	TP37	9,113,392	207.2 ± 39.0	1,888,081,563
	TP48	3,720,696	227.8 ± 40.5	847,609,750
	Soil Bulk	4,807,489	222.3 ± 40.6	1,068,497,871

“Bulk” samples are the unsheared bulks. “Phos.” samples are the phosphatase-treated unsheared bulks. “Sheared” samples are the sheared bulks. The “No. Filtered Sequences” is the number of sequences after filtering for sequencing artifacts (e.g. PCR duplicates). “Molecular Length” is the mean length (± standard deviation) of the generated reads.

### Illumina HiSeq 2500 sequencing

Initial tSMS results (see below) revealed very high microbial species diversity in the bone and soil samples. The very short sequence length hampered accurate identification of these species. Therefore, we constructed Illumina libraries from six bone extracts representing single individuals and the bulk soil sample using the PrepX Illumina Kit (IntegenX) on the Apollo 324 according to manufacturer’s instructions using NEXTflex™ DNA barcodes (BioO Scientific). The indexed libraries were then enriched by 13 cycles of PCR using the NEXTflex™ kit. Library qualities were confirmed via fluorometric quantitation (Qubit®), analysis on a high-specificity DNA chip (Agilent 2100) and quantitative PCR using the KAPA Library Quantification Kit – Illumina/Universal (KAPA Biosystems). The libraries were then pooled in equimolar ratios, and paired-end 150 bp reads were generated on the Illumina HiSeq 2500 platform. Initial quality control and demultiplexing was performed using CASAVA 1.8.2. Paired-end reads were merged using PANDAseq 2.4.0 [34]. Adapter artifacts and PCR duplicates were removed using TagDust 1.12 [35] and CD-HIT 4.6 [36]. The library sequencing qualities were checked using FastQC 1.32 [37]. The final data sets contained 3.7–9.6 million quality-controlled reads per library (Table 2).

### Bioinformatic analysis

Sequence file formats were manipulated using SAMtools 0.1.18 [38]. Sequence data sets were aligned against reference genomes of interest (Additional file 1, see Results and discussion below) using BWA 0.6.2 [39,40] according to the recommended settings in [41]. Sequences were also aligned against the GenBank® non-redundant nucleotide database using megaBLAST (BLAST 2.2.25+) [42] and analyzed in MEGAN 4.70.4 [43]. Statistical analyses were performed in R 2.15.3 [44] or using Biopieces [45] and custom scripts.

## Results and discussion

### Human DNA and authenticity

Alignment of the samples against the human (*Homo sapiens*) reference genome (GRCh37.p11) revealed that 0.66%–6.40% of the HeliScope reads in each channel and 0.00%–0.42% of Illumina sequences in each library corresponded to human DNA (Table 3, Additional file 1). The HeliScope endogenous human DNA quantifications are probably an underestimate of the true human DNA content since short sequences with low information contents are unlikely to map against the reference genome with sufficient confidence to be assigned (See ‘Soil Complexity’ below). Furthermore, the mean lengths (~25 bp) of the sequences mapped to the human genome were shorter than those (~34 bp) of all the sequences (mapped and unmapped included) in each channel (one-tailed *t*-test, *p* < 0.00001, Tables 2 and 3). As in [32], analysis of the mapped reads’ substitution artifacts using mapDamage2.0 [46] showed a uniform distribution of C → T and G → A transitions along the molecules. These substitutions typically accumulate at the 5′- and 3′-termini of ancient DNA molecules respectively [47]. These results suggest that the HeliScope chemistry cannot sequence through uracil lesions effectively, thereby shortening the recovered sequences of the endogenous ancient molecules and producing a uniform transition profile.

**Table 3:** Alignment statistics of reads mapped against the human genome

**Platform**	**Sample Description**	**Reads Mapped (% Mapped)**	**Molecular Length (bp)**	**Total Mapped (bp)**
HeliScope	Grand Plaza Bulk 1	254,977 (0.82%)	25.2 ± 2.7	6,414,294
	Grand Plaza Bulk 2	283,309 (0.77%)	25.2 ± 2.8	6,013,180
	Grand Plaza Phos. 1	186,016 (0.71%)	25.1 ± 2.5	4,668,531
	Grand Plaza Phos. 2	152,275 (0.66%)	25.2 ± 2.7	3,838,807
	Churchyard Bulk 1	387,992 (1.34%)	27.3 ± 5.3	10,579,724
	Churchyard Bulk 2	382,493 (1.41%)	27.3 ± 5.3	10,425,423
	Churchyard Phos. 1	250,427 (0.88%)	26.2 ± 4.3	6,559,094
	Churchyard Phos. 2	312,392 (1.05%)	26.5 ± 4.7	8,290,314
	Grand Plaza Sheared	254 (6.21%)	24.5 ± 1.0	6,226
	Churchyard Sheared	340 (6.40%)	24.5 ± 0.8	8,325
	Soil Bulk	9,441 (1.12%)	24.9 ± 2.1	234,694
	Soil Sheared	2,895 (2.35%)	24.8 ± 1.9	71,816
HiSeq 2500	TP04	2,655 (0.03%)	214.4 ± 39.6	569,144
	TP09	27,430 (0.41%)	192.1 ± 36.4	5.267,951
	TP10	1,332 (0.02%)	204.9 ± 37.6	272,903
	TP18	5,521 (0.00%)	201.7 ± 35.4	1,113,561
	TP37	6,172 (0.00%)	191.0 ± 31.4	1,179,076
	TP48	300 (0.00%)	208.6 ± 39.3	62,568
	Soil Bulk	37 (0.00%)	181.2 ± 34.0	6,706

“Bulk” samples are the unsheared bulks. “Phos.” samples are the phosphatase-treated unsheared bulks. “Sheared” samples are the sheared bulks. “% Mapped” indicates the percentage of the total number of reads comprised by the reads mapped onto the human genome. “Molecular Length” is the mean length (± standard deviation) of the mapped reads.

Due to the HeliScope sequences’ short read lengths, false positives due to the presence of contamination from closely related species (e.g. rodents) were a concern. Therefore, we also aligned the HeliScope data sets against the rat (*Rattus norvegicus*) genome (Rnor_5.0) and the chimpanzee (*Pan troglodytes*) genome (Pan_troglodytes-2.1.4) (Additional file 1). This ad-hoc test revealed a significant gradient of increasing numbers of hits against the genomes more closely related to humans (Kruskal-Wallis test, *p* = 0.0004609) with a concomitant increase in mean hit length in the more closely related species (Kruskal-Wallis test, *p* = 0.007629), suggesting that the HeliScope human-aligned reads derived from humans (Additional file 2).

Human DNA (1.12%–2.35% of all reads) was also identified in the soil HeliScope data sets, raising the possibility of laboratory contamination affecting our data. Nevertheless, analysis of the same soil bulk sample via Illumina sequencing revealed only 37 sequences (0.00%) that matched humans in the soil at the longer lengths (150–298 bp) obtained in the Illumina data. Comparatively, the individual bones had 300–27,430 human DNA sequences (0.01–0.42%) at equivalent lengths (Table 3). This indicates that the human DNA in the soil has undergone more degradation than that in the bone, a result inconsistent with the laboratory contamination hypothesis [30]. Moreover, although mitochondrial haplotype profiles were incomplete due to absence of a mitochondrial DNA enrichment step, the individual haplotypes (based on the Illumina sequences) generally agreed with those found in [29], although a high rate of nucleotide misincorporations was observed (Additional file 3). Analysis of these substitutions using mapDamage2.0 showed the characteristic ancient DNA pattern, with higher rates of C → T and G → A transitions at the 5′- and 3′-termini respectively [47]. This suggests that the human DNA in the bone samples is endogenous. It is possible that the soil human DNA derives from archaeologists in the field, although this is relatively unlikely since the bones and soil were collected while wearing gloves. Since the site was abandoned shortly after the *huey cocoliztli* pandemic [27], the most likely source of human DNA in the soil is not from later occupants of the site, but rather the numerous decomposing dead (estimated to at least 800 individuals) interred nearly simultaneously in the Grand Plaza cemetery. Detection of leached DNA is especially likely since the soil samples were collected immediately adjacent to the interred skeletons. If endogenous human DNA leached into the soil, it is possible that DNA from the *huey cocoliztli* pathogen may also have. However, since pathogen DNA is typically much rarer than host DNA [e.g. [13], this level of DNA leaching would probably be undetectable using current methodologies.

### Candidate diseases

Based on historical descriptions of the *huey cocoliztli*, likely candidate pathogens include pneumonic plague (*Yersinia pestis*), typhus and other forms of rickettsiosis (*Rickettsia* spp.), smallpox or alastrim (*Variola* spp.), and viral hemorrhagic fevers (VHF), such as Dengue and Yellow Fever [26,28,48,49]. Measles (*Morbillivirus Measles virus*), dysentery, influenza (*Influenzavirus* spp.), pneumonia and pleurisy have also been suggested, although these are less likely diagnoses since the historical descriptions of the *huey cocolitzli* symptoms do not match these diseases’ extant presentations [28]. Since dysentery, pneumonia and pleurisy can be caused by a wide range of organisms and are unlikely diagnoses for the *huey cocoliztli*, we did not analyze them further via the candidate genome approach described here. Additionally, VHFs, measles and influenza are caused by RNA viruses, which are unlikely to survive in the archaeological record due to RNA’s instability and the ubiquity of RNases [50]. Nevertheless, some have reported the successful amplification and sequencing of RNA viruses from medical archival preserved tissue dating back several decades [51,52], and Fordyce and colleagues [50] reported high-throughput sequencing of RNA transcripts in 700-year-old maize kernels. Moreover, the HeliScope will sequence RNA directly (even using DNA settings for the machine as conducted here albeit with reduced efficiency), so there remains a finite detection possibility for RNA viruses.

A substantial proportion of the unsheared HeliScope reads (0.01%, 2198–3880 reads per channel) matched the *Yersinia pestis* genome in both the Grand Plaza and Churchyard populations (Additional file 1). This also matched the proportion of putative *Y. pestis* reads found in the HeliScope-sequenced soil data set (0.01%, 63 reads) (Table 4). BLAST analysis of the mapped reads in MEGAN showed that these identifications were non-specific, with the sequences matching a wide variety of organisms, suggesting these mapped reads are false positives (Additional file 4). According to the BLAST identifications, only 0.29% and 2.0% of the reads mapped to the *Yerinia pestis* genome by BWA matched the Enterobacteriaceae and Gammaproteobacteria respectively, *Yersinia*’s family and class.

**Table 4:** **Alignment statistics of reads mapped against the *Yersinia pestis *** **genome**

**Platform**	**Sample Description**	**Reads Mapped (% Mapped)**	**Molecular Length (bp)**	**Total Mapped (bp)**
HeliScope	Grand Plaza Bulk 1	3,880 (0.01%)	28.8 ± 5.6	111,646
	Grand Plaza Bulk 2	3,451 (0.01%)	30.0 ± 5.7	99,916
	Grand Plaza Phos. 1	2,626 (0.01%)	28.5 ± 5.4	74,846
	Grand Plaza Phos. 2	2,198 (0.01%)	28.9 ± 5.8	63,479
	Churchyard Bulk 1	3,794 (0.01%)	28.8 ± 5.8	109,284
	Churchyard Bulk 2	3,672 (0.01%)	28.9 ± 5.9	106,060
	Churchyard Phos. 1	2,595 (0.01%)	28.1 ± 5.4	72,809
	Churchyard Phos. 2	3,723 (0.01%)	28.5 ± 5.7	106,284
	Grand Plaza Sheared	0 (0.00%)	—	0
	Churchyard Sheared	2 (0.04%)	24.0 ± 0.0	48
	Soil Bulk	63 (0.01%)	26.3 ± 3.7	1,656
	Soil Sheared	3 (0.00%)	25.3 ± 1.2	76
HiSeq 2500	TP04	0 (0.00%)	—	0
	TP09	0 (0.00%)	—	0
	TP10	0 (0.00%)	—	0
	TP18	0 (0.00%)	—	0
	TP37	0 (0.00%)	—	0
	TP48	0 (0.00%)	—	0
	Soil Bulk	0 (0.00%)	—	0

“Bulk” samples are the unsheared bulks. “Phos.” samples are the phosphatase-treated unsheared bulks. “Sheared” samples are the sheared bulks. “% Mapped” indicates the percentage of the total number of reads comprised by the reads mapped onto the *Yersinia pestis* genome. “Molecular Length” is the mean length (± standard deviation) of the mapped reads.

Alignment of the unsheared Churchyard and Grand Plaza bulk HeliScope data against typhus (*Rickettsia prowazekii* and *Rickettsia typhi*) genomes found that 0.00%–0.01% (1,210–2,089 reads) corresponded to these species (Table 5, Additional file 1). The identification of *Rickettsia* in bone matches its prevalence in the HeliScope-sequenced soil sample (0.00%, 30 reads). It is possible that the putative *Rickettsia* DNA in the soil derives from leaching from the bone. However, this explanation is improbable since the quantity of leached pathogen DNA is expected to be too small to detect given the low amounts of soil human DNA [e.g. [13]. Moreover, there were no differences between the Grand Plaza and Churchyard graveyards in terms of these species’ prevalences or lengths of the aligned sequences (30 ± 6 bp). Only one read corresponding to *Rickettsia* was found in the Illumina data sets. BLAST analysis of the *Rickettsia* hits showed that these sequences derived from various soil organisms rather than *bona fide* pathogens (Additional file 5). According to BLAST analyses, no reads matched the Rickettsiales and only 1.3% belonged to the Alphaproteobacteria order. We thus find no evidence that rickettsiosis is responsible for the *huey cocoliztli*.

**Table 5:** Alignment statistics of reads mapped against typhus genomes

**Platform**	**Sample Description**	**Reads Mapped (% Mapped)**	**Molecular Length (bp)**	**Total Mapped (bp)**
HeliScope	Grand Plaza Bulk 1	2,089 (0.01%)	30.2 ± 5.9	63,008
	Grand Plaza Bulk 2	1,901 (0.01%)	30.4 ± 6.1	57,799
	Grand Plaza Phos. 1	1,400 (0.00%)	30.3 ± 5.8	42,381
	Grand Plaza Phos. 2	1,142 (0.00%)	30.7 ± 6.2	35,032
	Churchyard Bulk 1	1,998 (0.01%)	29.8 ± 6.0	59,602
	Churchyard Bulk 2	1,905 (0.01%)	30.1 ± 6.2	57,251
	Churchyard Phos. 1	1,210 (0.00%)	29.9 ± 6.0	36,209
	Churchyard Phos. 2	1,754 (0.01%)	30.2 ± 6.2	53,055
	Grand Plaza Sheared	2 (0.05%)	24.5 ± 0.7	49
	Churchyard Sheared	0 (0.00%)	—	0
	Soil Bulk	30 (0.00%)	27.0 ± 4.5	810
	Soil Sheared	4 (0.00%)	24.3 ± 0.5	97
HiSeq 2500	TP04	0 (0.00%)	—	0
	TP09	0 (0.00%)	—	0
	TP10	1 (0.00%)	156	156
	TP18	0 (0.00%)	—	0
	TP37	0 (0.00%)	—	0
	TP48	0 (0.00%)	—	0
	Soil Bulk	0 (0.00%)	—	0

“Bulk” samples are the unsheared bulks. “Phos.” samples are the phosphatase-treated unsheared bulks. “Sheared” samples are the sheared bulks. “% Mapped” indicates the percentage of the total number of reads comprised by the reads mapped onto the typhus genome. “Molecular Length” is the mean length (± standard deviation) of the mapped reads.

A negligible number (11–30 reads per channel in the unsheared HeliScope bone data sets, none in the Illumina data sets) of reads mapped onto the *Variola* genome, the one candidate DNA virus (Table 6, Additional file 1). Alignment against the RNA virus candidates, including all four primary families of VHF (Arenaviridae, Bunyaviridae, Filoviridae, and Flaviviridae), measles and influenza, also yielded negative results (<120 reads per channel per genome, Tables 7,8,9,10,11 and 12, Additional file 1).

**Table 6:** **Alignment statistics of reads mapped against the *Variola *** **virus genome**

**Platform**	**Sample Description**	**Reads Mapped (% Mapped)**	**Molecular Length (bp)**	**Total Mapped (bp)**
HeliScope	Grand Plaza Bulk 1	19 (0.00%)	24.2 ± 0.5	459
	Grand Plaza Bulk 2	28 (0.00%)	24.7 ± 0.9	692
	Grand Plaza Phos. 1	11 (0.00%)	24.3 ± 0.5	267
	Grand Plaza Phos. 2	14 (0.00%)	24.6 ± 1.2	344
	Churchyard Bulk 1	35 (0.00%)	24.9 ± 2.0	870
	Churchyard Bulk 2	30 (0.00%)	25.7 ± 5.4	772
	Churchyard Phos. 1	21 (0.00%)	25.5 ± 3.9	535
	Churchyard Phos. 2	28 (0.00%)	24.8 ± 2.1	693
	Grand Plaza Sheared	0 (0.00%)	—	0
	Churchyard Sheared	0 (0.00%)	—	0
	Soil Bulk	1 (0.00%)	24.0	24
	Soil Sheared	0 (0.00%)	—	0
HiSeq 2500	TP04	0 (0.00%)	—	0
	TP09	0 (0.00%)	—	0
	TP10	0 (0.00%)	—	0
	TP18	0 (0.00%)	—	0
	TP37	0 (0.00%)	—	0
	TP48	0 (0.00%)	—	0
	Soil Bulk	0 (0.00%)	—	0

“Bulk” samples are the unsheared bulks. “Phos.” samples are the phosphatase-treated unsheared bulks. “Sheared” samples are the sheared bulks. “% Mapped” indicates the percentage of the total number of reads comprised by the reads mapped onto the *Variola* genome. “Molecular Length” is the mean length (± standard deviation) of the mapped reads.

**Table 7:** Alignment statistics of reads mapped against Arenaviridae genomes

**Platform**	**Sample Description**	**Reads Mapped (% Mapped)**	**Molecular Length (bp)**	**Total Mapped (bp)**
HeliScope	Grand Plaza Bulk 1	37 (0.00%)	25.9 ± 3.4	959
	Grand Plaza Bulk 2	24 (0.00%)	26.9 ± 4.6	646
	Grand Plaza Phos. 1	13 (0.00%)	24.5 ± 0.7	319
	Grand Plaza Phos. 2	15 (0.00%)	26.5 ± 3.8	397
	Churchyard Bulk 1	193 (0.00%)	26.3 ± 3.7	5,071
	Churchyard Bulk 2	254 (0.00%)	26.3 ± 3.4	6,673
	Churchyard Phos. 1	65 (0.00%)	26.3 ± 3.7	1,711
	Churchyard Phos. 2	110 (0.00%)	26.8 ± 4.1	2,946
	Grand Plaza Sheared	0 (0.00%)	—	0
	Churchyard Sheared	0 (0.00%)	—	0
	Soil Bulk	2 (0.00%)	24. 5 ± 0.7	49
	Soil Sheared	0 (0.00%)	—	0
HiSeq 2500	TP04	0 (0.00%)	—	0
	TP09	0 (0.00%)	—	0
	TP10	0 (0.00%)	—	0
	TP18	0 (0.00%)	—	0
	TP37	0 (0.00%)	—	0
	TP48	0 (0.00%)	—	0
	Soil Bulk	0 (0.00%)	—	0

“Bulk” samples are the unsheared bulks. “Phos.” samples are the phosphatase-treated unsheared bulks. “Sheared” samples are the sheared bulks. “% Mapped” indicates the percentage of the total number of reads comprised by the reads mapped onto Arenaviridae genomes. “Molecular Length” is the mean length (± standard deviation) of the mapped reads.

**Table 8:** Alignment statistics of reads mapped against Bunyaviridae genomes

**Platform**	**Sample Description**	**Reads Mapped (% Mapped)**	**Molecular Length (bp)**	**Total Mapped (bp)**
HeliScope	Grand Plaza Bulk 1	8 (0.00%)	24.5 ± 0.9	196
	Grand Plaza Bulk 2	10 (0.00%)	24.4 ± 0.5	244
	Grand Plaza Phos. 1	6 (0.00%)	24.3 ± 0.8	146
	Grand Plaza Phos. 2	3 (0.00%)	25.0 ± 1.0	75
	Churchyard Bulk 1	10 (0.00%)	24.3 ± 0.5	243
	Churchyard Bulk 2	16 (0.00%)	24.3 ± 0.6	388
	Churchyard Phos. 1	10 (0.00%)	24.6 ± 0.5	246
	Churchyard Phos. 2	15 (0.00%)	24.6 ± 0.7	369
	Grand Plaza Sheared	0 (0.00%)	—	0
	Churchyard Sheared	0 (0.00%)	—	0
	Soil Bulk	0 (0.00%)	—	0
	Soil Sheared	0 (0.00%)	—	0
HiSeq 2500	TP04	0 (0.00%)	—	0
	TP09	0 (0.00%)	—	0
	TP10	0 (0.00%)	—	0
	TP18	0 (0.00%)	—	0
	TP37	0 (0.00%)	—	0
	TP48	0 (0.00%)	—	0
	Soil Bulk	0 (0.00%)	—	0

“Bulk” samples are the unsheared bulks. “Phos.” samples are the phosphatase-treated unsheared bulks. “Sheared” samples are the sheared bulks. “% Mapped” indicates the percentage of the total number of reads comprised by the reads mapped onto Bunyaviridae genomes. “Molecular Length” is the mean length (± standard deviation) of the mapped reads.

**Table 9:** Alignment statistics of reads mapped against Filoviridae genomes

**Platform**	**Sample Description**	**Reads Mapped (% Mapped)**	**Molecular Length (bp)**	**Total Mapped (bp)**
HeliScope	Grand Plaza Bulk 1	27 (0.00%)	24.4 ± 0.9	659
	Grand Plaza Bulk 2	25 (0.00%)	24.6 ± 0.8	614
	Grand Plaza Phos. 1	18 (0.00%)	24.7 ± 1.2	444
	Grand Plaza Phos. 2	15 (0.00%)	24.1 ± 0.3	361
	Churchyard Bulk 1	22 (0.00%)	24.2 ± 0.5	532
	Churchyard Bulk 2	26 (0.00%)	24.3 ± 0.6	631
	Churchyard Phos. 1	22 (0.00%)	24.4 ± 0.7	536
	Churchyard Phos. 2	23 (0.00%)	24.3 ± 0.5	560
	Grand Plaza Sheared	0 (0.00%)	—	0
	Churchyard Sheared	0 (0.00%)	—	0
	Soil Bulk	0 (0.00%)	—	0
	Soil Sheared	0 (0.00%)	—	0
HiSeq 2500	TP04	0 (0.00%)	—	0
	TP09	0 (0.00%)	—	0
	TP10	0 (0.00%)	—	0
	TP18	0 (0.00%)	—	0
	TP37	0 (0.00%)	—	0
	TP48	0 (0.00%)	—	0
	Soil Bulk	0 (0.00%)	—	0

“Bulk” samples are the unsheared bulks. “Phos.” samples are the phosphatase-treated unsheared bulks. “Sheared” samples are the sheared bulks. “% Mapped” indicates the percentage of the total number of reads comprised by the reads mapped onto Filoviridae genomes. “Molecular Length” is the mean length (± standard deviation) of the mapped reads.

**Table 10:** Alignment statistics of reads mapped against Flaviviridae genomes

**Platform**	**Sample Description**	**Reads Mapped (% Mapped)**	**Molecular Length (bp)**	**Total Mapped (bp)**
HeliScope	Grand Plaza Bulk 1	93 (0.00%)	24.6 ± 0.8	2,284
	Grand Plaza Bulk 2	111 (0.00%)	24.6 ± 0.9	2,726
	Grand Plaza Phos. 1	112 (0.00%)	24.7 ± 0.9	2,762
	Grand Plaza Phos. 2	76 (0.00%)	24.8 ± 1.0	1,881
	Churchyard Bulk 1	122 (0.00%)	24.6 ± 1.3	3,004
	Churchyard Bulk 2	126 (0.00%)	24.7 ± 1.1	3,116
	Churchyard Phos. 1	106 (0.00%)	24.5 ± 0.8	2,602
	Churchyard Phos. 2	105 (0.00%)	24.5 ± 0.8	2,573
	Grand Plaza Sheared	0 (0.00%)	—	0
	Churchyard Sheared	0 (0.00%)	—	0
	Soil Bulk	3 (0.00%)	24.3 ± 0.6	73
	Soil Sheared	0 (0.00%)	—	0
HiSeq 2500	TP04	0 (0.00%)	—	0
	TP09	0 (0.00%)	—	0
	TP10	0 (0.00%)	—	0
	TP18	0 (0.00%)	—	0
	TP37	0 (0.00%)	—	0
	TP48	0 (0.00%)	—	0
	Soil Bulk	0 (0.00%)	—	0

“Bulk” samples are the unsheared bulks. “Phos.” samples are the phosphatase-treated unsheared bulks. “Sheared” samples are the sheared bulks. “% Mapped” indicates the percentage of the total number of reads comprised by the reads mapped onto Flaviviridae genomes. “Molecular Length” is the mean length (± standard deviation) of the mapped reads.

**Table 11:** **Alignment statistics of reads mapped against the *Morbillivirus Measles virus *** **genome**

**Platform**	**Sample Description**	**Reads Mapped (% Mapped)**	**Molecular Length (bp)**	**Total Mapped (bp)**
HeliScope	Grand Plaza Bulk 1	1 (0.00%)	24.0	24
	Grand Plaza Bulk 2	1 (0.00%)	24.0	24
	Grand Plaza Phos. 1	2 (0.00%)	25.0 ± 1.4	50
	Grand Plaza Phos. 2	0 (0.00%)	—	0
	Churchyard Bulk 1	1 (0.00%)	24.0	72
	Churchyard Bulk 2	5 (0.00%)	24.0 ± 0.9	122
	Churchyard Phos. 1	1 (0.00%)	24.0	24
	Churchyard Phos. 2	3 (0.00%)	24. 0 ± 0.0	72
	Grand Plaza Sheared	0 (0.00%)	—	0
	Churchyard Sheared	0 (0.00%)	—	0
	Soil Bulk	0 (0.00%)	—	0
	Soil Sheared	0 (0.00%)	—	0
HiSeq 2500	TP04	0 (0.00%)	—	0
	TP09	0 (0.00%)	—	0
	TP10	0 (0.00%)	—	0
	TP18	0 (0.00%)	—	0
	TP37	0 (0.00%)	—	0
	TP48	0 (0.00%)	—	0
	Soil Bulk	0 (0.00%)	—	0

“Bulk” samples are the unsheared bulks. “Phos.” samples are the phosphatase-treated unsheared bulks. “Sheared” samples are the sheared bulks. “% Mapped” indicates the percentage of the total number of reads comprised by the reads mapped onto influenza genomes. “Molecular Length” is the mean length (± standard deviation) of the mapped reads.

**Table 12:** Alignment statistics of reads mapped against influenza genomes

**Platform**	**Sample Description**	**Reads Mapped (% Mapped)**	**Molecular Length (bp)**	**Total Mapped (bp)**
HeliScope	Grand Plaza Bulk 1	3 (0.00%)	24.0 ± 0.0	72
	Grand Plaza Bulk 2	5 (0.00%)	24.0 ± 0.0	120
	Grand Plaza Phos. 1	3 (0.00%)	24.0 ± 0.0	72
	Grand Plaza Phos. 2	0 (0.00%)	—	0
	Churchyard Bulk 1	3 (0.00%)	24.0 ± 0.0	72
	Churchyard Bulk 2	5 (0.00%)	24.0 ± 0.9	122
	Churchyard Phos. 1	3 (0.00%)	24.0 ± 0.0	72
	Churchyard Phos. 2	5 (0.00%)	24.0 ± 0.0	120
	Grand Plaza Sheared	0 (0.00%)	—	0
	Churchyard Sheared	0 (0.00%)	—	0
	Soil Bulk	0 (0.00%)	—	0
	Soil Sheared	0 (0.00%)	—	0
HiSeq 2500	TP04	0 (0.00%)	—	0
	TP09	0 (0.00%)	—	0
	TP10	0 (0.00%)	—	0
	TP18	0 (0.00%)	—	0
	TP37	0 (0.00%)	—	0
	TP48	0 (0.00%)	—	0
	Soil Bulk	0 (0.00%)	—	0

“Bulk” samples are the unsheared bulks. “Phos.” samples are the phosphatase-treated unsheared bulks. “Sheared” samples are the sheared bulks. “% Mapped” indicates the percentage of the total number of reads comprised by the reads mapped onto influenza genomes. “Molecular Length” is the mean length (± standard deviation) of the mapped reads.

In conclusion, alignment of the HeliScope and Illumina data sets compared against the candidate disease reference genomes produced inconclusive results. While it is possible that the *huey cocoliztli* is not preserved in femoral cortical bone, historical documents describe a systemic infection with symptoms including hemorrhage and ulceration in multiple organs [48]. It is therefore likely that the pathogen would be preserved in all vascularized tissues. Additionally, due to the instability of the RNA molecule and the paucity of the virus sequence databases, negative viral results are currently difficult to evaluate. Our results demonstrate that false positives are a serious problem for analyses identifying molecules via alignment against reference genomes and for analyses that omit sequencing archaeological controls. Nevertheless, capture of complete species-specific diagnostic sequences and genomes (e.g. [13,14]) may be a viable method for isolating and verifying ancient pathogen DNA in the absence of these controls.

Currently, few high-throughput palaeopathogen DNA analyses have been conducted. The majority of studies have been PCR-based, an approach whose limitations are well documented [e.g. [21-23]. Although it is difficult to compare different archaeological collections and data sets in terms of the likelihood of ancient pathogen recovery, our data are illustrative of the challenges encountered in high-throughput metagenomic pathogen studies. They emphasize the level of analytic rigor and proof required to authenticate ancient pathogen analyses. In light of our findings, some previous metagenomic ancient DNA research claiming the discovery of pathogen-derived DNA may need further verification. For instance, although Thèves and colleagues [22] and Khairat and colleagues [53] report identifying *Bordetella sp*., *Streptococcus pneumoniae* and *Shigella dystenteriae*[22] and *Plasmodium falciparum* and *Toxoplasma gondii*[53] respectively, neither group sequenced archaeological controls such as soil or mummy wrappings. Thèves and colleagues amplified and sequenced microbial barcode 16S genes, increasing their results’ reliability since these genes are well characterized across a wide variety of species. Khairat and colleagues, however, based their apicomplexan identifications on MEGAN analysis, with no attempt to evaluate the species-specificity of the sequenced molecules. These identifications are therefore suspect since the genetic databases are skewed towards pathogenic members of this lineage. Additionally, a recent study by Chan and colleagues [54] claiming the identification of multiple strains of pathogenic tuberculosis (*Mycobacterium tuberculosis*) through non-targeted metagenomic sequencing has demonstrated insufficient analytical rigor to support their conclusions. The authors aligned their sequences against a single strain of pathogenic tuberculosis, but did not account for misalignments or environmental contamination with ubiquitous soil mycobacteria. Chan and colleagues’ data merit reanalysis with appropriate environmental controls. We recommend that the authors of these three studies demonstrate the veracity of their findings using a targeted capture approach and further bioinformatic analysis.

### Soil complexity

Examination of the Teposcolula soil DNA in MEGAN revealed that the microenvironment is complex, making identification of species-of-interest difficult. While there were significant differences in the relative frequencies of microorganisms between the Grand Plaza and Churchyard samples (Additional file 6), these differences corresponded to variation in the distribution of environment-derived organisms across the site rather than pathogen-related frequency variation. For instance, we found significant differences in the prevalence of Viridiplantae and the Rhizobiaceae, which are almost certainly environmental contaminants.

Moreover, the vast majority of HeliScope sequences (~95%) are unknown due to both limitations in the databases and the non-specificity of the short sequences. Due to the limitations of the genetic databases, some authors have amplified 16S genes (the most studied microbial barcode genes) before high-throughput sequencing in order to derive more species-informative ancient DNA metagenomic data sets (e.g. [24,55]). We have not conducted this PCR procedure here since it negates the advantages of single-molecule sequencing. Instead, since the majority of sequences in the soil Illumina data set are unknown, we attempted to use the Illumina data set as a pseudo-‘reference metagenome’ to identify environmental contaminants in our HeliScope data. However, only 1.13%–9.62% of the sequences in the Helicos data sets (including the HeliScope soil sequence pools) mapped onto the Illumina soil sequences (Table 13). Given our low estimate of endogenous human DNA, we would expect >99% of the HeliScope reads (or 100% in the case of the soil reads) to map onto the Illumina soil data set. This indicates that the HeliScope is generating sequences with too low an information content to align against reference genomes with sufficient precision to be considered likely matches.

**Table 13:** Alignment statistics of HeliScope reads mapped against the Teposcolula Yucundaa soil Illumina-sequenced sample

**Sample Description**	**Reads Mapped (% Mapped)**	**Molecular Length (bp)**	**Total Mapped (bp)**
Grand Plaza Bulk 1	2,145,104 (6.88%)	28.6 ± 5.8	61,255,930
Grand Plaza Bulk 2	2,072,270 (6.73%)	28.6 ± 5.8	59,267,370
Grand Plaza Phos. 1	1,641,432 (6.25%)	28.1 ± 5.5	46,147,661
Grand Plaza Phos. 2	1,275,863 (5.53%)	28.5 ± 5.7	36,333,528
Churchyard Bulk 1	2,423,489 (8.37%)	29.4 ± 6.1	71,205,930
Churchyard Bulk 2	2,292,885 (8.48%)	29.4 ± 6.1	67,327,090
Churchyard Phos. 1	2,033,593 (7.15%)	28.7 ± 5.9	58,421,595
Churchyard Phos. 2	2,563,785 (8.64%)	28.9 ± 5.9	74,145,815
Grand Plaza Sheared	46 (1.13%)	24.5 ± 0.7	1,126
Churchyard Sheared	90 (1.70%)	24.6 ± 1.0	2,212
Soil Bulk	80,900 (9.62%)	29.0 ± 5.7	2,349,874
Soil Sheared	6,729 (5.46%)	27.1 ± 4.4	182,463

“Bulk” samples are the unsheared bulks. “Phos.” samples are the phosphatase-treated unsheared bulks. “Sheared” samples are the sheared bulks. “% Mapped” indicates the percentage of the total number of reads comprised by the reads mapped onto influenza genomes. “Molecular Length” is the mean length (± standard deviation) of the mapped reads.

### DNA length, GC content and sequencing yields

Agilent 2100 Bioanalyzer profiles of the unsheared DNA extracts revealed the typical bimodal distribution of molecular lengths, with a large primary peak between 1000 and 10,000 bp and a small secondary peak around 70 bp. These peaks have previously been determined to correspond primarily to microbial contamination (large peak) and a mixture of fragmented contaminant and authentic ancient DNA (small peak) respectively [20,32,56]. Shearing the extracts produced unimodal distributions with modal lengths between 150 and 200 bp.

Shearing of the DNA extracts reduced the total sequence yield drastically (5400–6800-fold reduction in the bone and 6.8-fold reduction in the soil), but yielded a concomitant 8.0–10.6-fold enrichment in the proportion of sequences mapping to the human genome in the bone samples and a 2.3-fold enrichment in human sequences in the soil. The cause of this yield reduction remains unclear. Variation between runs cannot completely explain it since the sheared and unsheared soil samples were sequenced on the same chip and samples from unrelated projects on the same chip had typical HeliScope sequencing yields [D. Jones, Pers. Comm. 2013]. Hypothetically, oxidative products (such as 8-oxoguanine and abasic sites) near the 3′-terminus of the sheared DNA molecule could disrupt the downstream poly (A)-tailing reaction and reduce the overall sequencing yield. Costello and colleagues [57] noted that shearing DNA samples to 150 bp lengths using the Covaris instrument produced 8-oxoguanine lesions yielding C → A transversion artifacts in downstream Illumina library production. Nevertheless, 8-oxoguanine lesions were rare and were only observable in libraries constructed from small initial DNA inputs. 8-oxoguanine lesions thus seem an implausible explanation for our results, although the production of abasic sites at the 3′-terminus cannot be ruled out.

An alternative explanation is that the shearing caused a decrease in the percentage of denaturable DNA molecules. DNA molecules that cannot be rendered single-stranded are unsequenceable on the HeliScope platform, thus reducing the effective input DNA concentration. One mechanism could be a relative increase in GC-rich sequences in the sheared samples, thus increasing the energy required for denaturation of these molecules. Shearing longer GC-rich DNAs into smaller and more numerous molecules would increase the apparent GC content since this would raise the relative number of available 3′-termini from the GC-rich sequences while the shorter AT-rich sequences would not shear into as many sequenceable fragments. Although the HeliScope platform is noted for its improved performance in both GC- and AT-rich regions in comparison to other high-throughput technologies, it is not impervious to these biochemical effects. This would cause an asymmetric denaturation step with only shorter and/or AT-rich molecules becoming single-stranded in the pool. An alternative, similar mechanism is that the sheared DNA molecules (which mostly derive from the high-molecular-weight peak) are more likely to be cross-linked to proteins and themselves, thus preventing denaturation and sequencing. Since the shearing raised the average length of the molecules (in comparison to the smaller ‘ancient DNA’ peak), this indicates that a far greater percentage of the sequenced molecules in the sheared pools were derived from the high-molecular-weight ‘microbial contaminant’ peak than from the ‘ancient DNA’ peak. Many microbial metagenomes have been noted to be particularly GC-rich. Our samples have a high GC content at baseline (mean 62.4%, standard deviation 1.1% in the unsheared bone and mean 62.1% in the soil). Shearing reduced the apparent GC content to 46.4% (standard deviation 1.0%) in the bones and a 58.1% in the soil. Moreover, there is a decrease in molecular length in comparison to their unsheared counterparts (median lengths of 26–30 bp in the sheared samples versus 32–34 bp in the unsheared samples). The AT enrichment, reduction in molecular length and increased percentage of human molecules are consistent with the denaturation hypothesis since the sequenced sheared molecules would on average have lower GC contents and be shorter, thus increasing the likelihood of sequencing endogenous molecules.

### Antarctic phosphatase treatment

Ginolhac and colleagues [32] reported a 7.5–9.7-fold enrichment in the total sequence yield in their tSMS data after treatment of samples with Antarctic phosphatase. Conversely, we observed no such enrichment in our data; phosphatase-treated samples had 80%–104% relative yield of their untreated counterparts. They also documented a decrease in the proportion of endogenous sequences in the phosphatase-treated samples (34%–50% relative yield compared to untreated samples). In general agreement with Ginolhac and colleagues’ results, we observed a decrease in endogenous human sequences after phosphatase treatment (70%–90% relative yield). The discrepancy in overall yields may be due to differences in the concentrations of sequenceable molecules in the DNA extracts, such as those possibly caused by varying burial environments (Pleistocene permafrost versus Mexican highlands), GC contents between the sample (~40% in their data versus ~60% in our individuals) and extraction procedures. If our samples had greater concentrations of sequenceable molecules (which is likely since our untreated sample yields exceeded those of Ginolhac and colleagues by ~100-fold), it is possible that we saturated the channel and thus limited the effects of phosphatase treatment. The discrepancy between Ginolhac and colleagues’ and our phosphatase treatment results could also be a statistical artifact. Due to the large number (typically 10–20 million) of sequences generated in each HeliScope channel and the significant variation between replicate runs (relative standard deviations ranged between 0.66% and 6.5%), the balance of effect sizes versus discriminatory power is difficult to optimize.

## Conclusions

Environmental contamination is a critical issue in ancient DNA investigations of diseases. Both false positives and false negatives are a serious problem. Previous investigations of ancient pathogens using both PCR and high-throughput sequencing may need to be re-evaluated because pathogen identifications require extensive verification procedures due to the high risk of false positives. Currently, molecular enrichment to isolate molecules of interest from the complex background (via either hybridization to probes or amplification of 16S regions) is the most promising route for ancient pathogen studies.

Furthermore, although the benefits of single-molecule sequencing are promising, methodological challenges remain in its application to ancient DNA research. We found that tSMS is not immune to GC-content-related biases and that the benefits of phosphatase-treatment are not universal. Analysis of these data remains complex due to the short lengths of the sequenced molecules and limitations of public databases. Bioinformatic methods developed for modern DNA analysis and for longer sequences are not appropriate for HeliScope ancient DNA data.

Finally, further methodological development is required for the identification of ancient pathogens to become routine and reliable. Until these techniques become available, the burden-of-proof is on the researchers reporting the discovery of these disease agents to demonstrate their results’ authenticity.

### Availability of supporting data

The data sets supporting the results of this article are available in the National Center for Biotechnology Information Sequence Read Archive, [SRP022977: http://www.ncbi.nlm.nih.gov/sra].

## Abbreviations

ADNA: Ancient deoxyribonucleic acid; BP: Base pairs; DNA: Deoxyribonucleic acid; PCR: Polymerase chain reaction; RNA: Ribonucleic acid; TSMS: True single-molecule sequencing; VHF: Viral hemorrhagic fever.

## Competing interests

The authors declare that they have no competing interests.

## Authors’ contributions

MGC conducted the molecular genetic analyses, performed the bioinformatic analysis and drafted the manuscript. NRG provided samples and conceived the project. FJR participated in the coordination and design of the project. NT performed molecular experiments and conceived, coordinated and led the project. All authors read and approved the final manuscript.

## Supplementary Material

Additional file 1 GenBank accessions for the genomes utilized in this study. Click here for file

Additional file 2 Alignment statistics of the HeliScope data sets against the rat and chimpanzee genomes. Click here for file

Additional file 3 Concordance of the mitochondrial haplotypes between our Illumina data sets and those reported in Warinner. Click here for file

Additional file 4 **MEGAN analysis of the reads mapped to the *Yersinia pestis *** **genome.** The reads are non-specific for the pathogen. Click here for file

Additional file 5 **MEGAN analysis of the reads mapped to *Rickettsia *** **genomes.** The reads are non-specific for the pathogens. Instead, they correspond to common environmental organisms. Click here for file

Additional file 6 **MEGAN comparison of the BLAST hits between the Grand Plaza (blue) and Churchyard (red) populations.** Significant differences in relative species prevalence are highlighted in black. The vast majority of the DNA sequences are derived from the environment, obscuring any ancient pathogen signal. Click here for file
