# Supplementary material for: False positives complicate ancient pathogen identifications using high-throughput shotgun sequencing
Source: BMC Res Notes. 2014 Feb 25;7:111. doi: 10.1186/1756-0500-7-111 (PMC3938818; doi:10.1186/1756-0500-7-111)
Supplement: Additional file 1 — GenBank accessions for the genomes utilized in this study. [file 1756-0500-7-111-S1.docx]

Additional file 1**:** GenBank accessions for the genomes utilized in this study

*Homo sapiens*: GCA_000001405.12

*Rattus norvegicus*: GCA_000001895.3

*Pan troglodytes*: GCA_000001515.4

*Yersinia pestis*: NC_008120.1–NC_008122.1, NC_008150.1

*Rickettsia* spp.: NC_000963.1, NC_006142.1

*Variola*: NC_001611.1

Arenaviridae: EF529744.1-EF529747.1, FJ805377.1-FJ805380.1, GQ862981.1, GQ862982.1, HQ126698.1-HQ126701.1, HQ688672.1-HQ688675.1, J04324.1, JF799977.1-JF799984.1, NC_004296.1,NC_005077.1- NC_005079.1-NC_005082.1, NC_005897.1, NC_006313.1, NC_006317.1, NC_006439.1, NC_006447.1, NC_006572.1-NC_006575.1, NC_010701.1, NC_0101756.1, NC_013057.1, NC_013058.1, NC_016152.1, NC_016153.1

Bunyaviridae: AB000851.1-AB000870.1, AB000872.1, AB000873.1, EF485030.1- EF485038.1, EU203678.2, EU262553.1, EU294510.1, EU879063.2, FJ94305.1-FJ943510.1, GQ118699.1, GQ118700.1, GU591164.1- GU591169.1, HM007350.1-HM007358.1, HM036208.1-HM036219.1, HM27176.1-HM627178.1, JN157805.1, K00610.1, K02539.1, M19420.1, NC_004108.1-NC_004110.1, NC_005776.1, NC_005777.1, NC_009896.1, U12396.1, U18979.1, U18980.1

Filoviridae: AB050936.1, AF086833.2, AF272001.1, AF499101.1, AF522874.1, AY142960.1, AY354458.1, AY430365.1, AY430366.1, AY769362.1, AY729654.1, DQ217792.1, DQ447649.1-DQ447660.1, EF446131.1, EF446132.1, EU224440.2, EU338380.1, EU500826.1-EU500828.1, FJ217161.1, FJ217162.1, FJ621583.1-FJ621585.1, FJ750953.1-FJ750959.1, FJ968794.1, HQ613402.1, HQ613403.1, JF828358.1, JN408064.1, JQ352763.1, NC_001608.3, NC_004161.1, NC_006432.1, NC_014373.1, NC_016144.1

Flaviviridae: NC_000943.1, NC_001437.1, NC_001474.2, NC_001475.2, NC_001477.1, NC_001563.2, NC_001564.1, NC_001672.1, NC_001809.1, NC_002031.1, NC_002640.1, NC_003635.1, NC_003675.1, NC_003676.1, NC_003687.1, NC_003690.1, NC_003996.1, NC_004119.1, NC_004355.1, NC_005039.1, NC_005062.1, NC_005064.1, NC_006551.1, NC_006947.1, NC_007580.2, NC_008604.2, NC_008718.1, NC_008719.1, NC_009026.2, NC_009028.2, NC_009029.2, NC_009942.1, NC_012532.1-NC_012534.1, NC_012671.1, NC_012735.1, NC_012932.1, NC_015843.2, NC_016958.1, NC_016997.1

Influenza: PRJNA14656,PRJNA15055,PRJNA15617
