# Supplementary material for: False positives complicate ancient pathogen identifications using high-throughput shotgun sequencing
Source: BMC Res Notes. 2014 Feb 25;7:111. doi: 10.1186/1756-0500-7-111 (PMC3938818; doi:10.1186/1756-0500-7-111)
Supplement: Additional file 2 — Alignment statistics of the HeliScope data sets against the rat and chimpanzee genomes. [file 1756-0500-7-111-S2.docx]

Additional file 2: Alignment statistics of HeliScope reads mapped against the rat (*Rattus norvegicus*) genome.

| Sample Description | Reads Mapped (% Mapped) | Molecular Length (bp) | Total Mapped (bp) |
| --- | --- | --- | --- |
| Grand Plaza Bulk 1 | 212,901 (0.68%) | 24.8 ± 1.8 | 5,275,557 |
| Grand Plaza Bulk 2 | 199,665 (0.65%) | 24.9 ± 2.0 | 4,974,320 |
| Grand Plaza Phos. 1 | 155,488 (0.59%) | 24.9 ± 1.9 | 3,862,790 |
| Grand Plaza Phos. 2 | 128,003 (0.56%) | 24.9 ± 1.9 | 3,187,048 |
| Churchyard Bulk 1 | 256,990 (0.89%) | 25.2 ± 2.8 | 6,470,164 |
| Churchyard Bulk 2 | 256,517 (0.95%) | 25.3 ± 2.9 | 6,487,959 |
| Churchyard Phos. 1 | 187,705 (0.66%) | 25.0 ± 2.5 | 4,696,295 |
| Churchyard Phos. 2 | 219,112 (0.74%) | 24.9 ± 2.4 | 5,465,319 |
| Grand Plaza Sheared | 270 (6.60%) | 24.4 ± 0.7 | 6,577 |
| Churchyard Sheared | 342 (6.44%) | 24.3 ± 0.7 | 8,342 |
| Soil Bulk | 8,448 (1.00%) | 24.7 ± 1.6 | 208,364 |
| Soil Sheared | 2,876 (2.33%) | 24.6 ± 1.2 | 71,701 |

“Bulk” samples are the unsheared bulks. “Phos.” samples are the phosphatase-treated unsheared bulks. “Sheared” samples are the sheared bulks. “% Mapped” indicates the percentage of the total number of reads comprised by the reads mapped onto the rat genome. “Molecular Length” is the mean length (± standard deviation) of the mapped reads.

Alignment statistics of HeliScope reads mapped against the chimpanzee (*Pan troglodytes*) genome.

| Sample Description | Reads Mapped (% Mapped) | Molecular Length (bp) | Total Mapped (bp) |
| --- | --- | --- | --- |
| Grand Plaza Bulk 1 | 243,519 (0.78%) | 25.1 ± 2.6 | 6,118,106 |
| Grand Plaza Bulk 2 | 226,656 (0.74%) | 25.2 ± 2.7 | 5,738,601 |
| Grand Plaza Phos. 1 | 177,236 (0.67%) | 25.1 ± 2.4 | 4,445,544 |
| Grand Plaza Phos. 2 | 145,229 (0.63%) | 25.2 ± 2.6 | 3,657,804 |
| Churchyard Bulk 1 | 366,434 (1.27%) | 27.1 ± 5.2 | 9,936,546 |
| Churchyard Bulk 2 | 362,474 (1.34%) | 27.1 ± 5.1 | 9,826,483 |
| Churchyard Phos. 1 | 236,667 (0.83%) | 26.1 ± 4.2 | 6,175,378 |
| Churchyard Phos. 2 | 295,165 (1.00%) | 26.4 ± 4.6 | 7,797,452 |
| Grand Plaza Sheared | 245 (5.99%) | 24.5 ± 1.0 | 6,006 |
| Churchyard Sheared | 328 (6.18%) | 24.5 ± 0.8 | 8,028 |
| Soil Bulk | 9,042 (1.08%) | 24.9 ± 2.1 | 225,722 |
| Soil Sheared | 2,811 (2.28%) | 24.8 ± 1.9 | 69,722 |

“Bulk” samples are the unsheared bulks. “Phos.” samples are the phosphatase-treated unsheared bulks. “Sheared” samples are the sheared bulks. “% Mapped” indicates the percentage of the total number of reads comprised by the reads mapped onto the chimpanzee genome. “Molecular Length” is the mean length (± standard deviation) of the mapped reads.
