# Supplementary material for: False positives complicate ancient pathogen identifications using high-throughput shotgun sequencing
Source: BMC Res Notes. 2014 Feb 25;7:111. doi: 10.1186/1756-0500-7-111 (PMC3938818; doi:10.1186/1756-0500-7-111)
Supplement: Additional file 3 — Concordance of the mitochondrial haplotypes between our Illumina data sets and those reported in Warinner. [file 1756-0500-7-111-S3.docx]

Additional file 3**:** Concordance of the mitochondrial haplotypes between our Illumina data sets and those reported in Warinner**.**

| Site | TP09 PCR | TP09 Illumina | TP18 PCR | TP18 Illumina | TP37 PCR | TP37 Illumina |
| --- | --- | --- | --- | --- | --- | --- |
| 663 | G | G (1) | A | — | A | — |
| 5178 | — | — | C | T* | C | — |
| 8281-8289 | No Deletion | No Deletion | Deletion | — | Deletion | — |
| 10398 | A | A (2) | C | — | A | A |
| 10400 | C | C/T* (2) | A | — | C | C |
| 13263 | A | A | A | — | A | — |

*Note that these discrepancies are explainable by C→T transition errors due to the low sequencing depth. Contamination cannot be ruled out, but the concordance of the other SNPs indicates that this would be at a low level.

**Reference**

1. Warinner CG: **Life and death at Teposcolula Yucundaa: mortuary, archaeogenetic, and isotopic investigations of the early colonial period in Mexico.** *PhD thesis.* Harvard University, Human Evolutionary Biology Department; 2010.
