# Supplementary figures and images for: False positives complicate ancient pathogen identifications using high-throughput shotgun sequencing
Source: BMC Res Notes. 2014 Feb 25;7:111. doi: 10.1186/1756-0500-7-111 (PMC3938818; doi:10.1186/1756-0500-7-111)

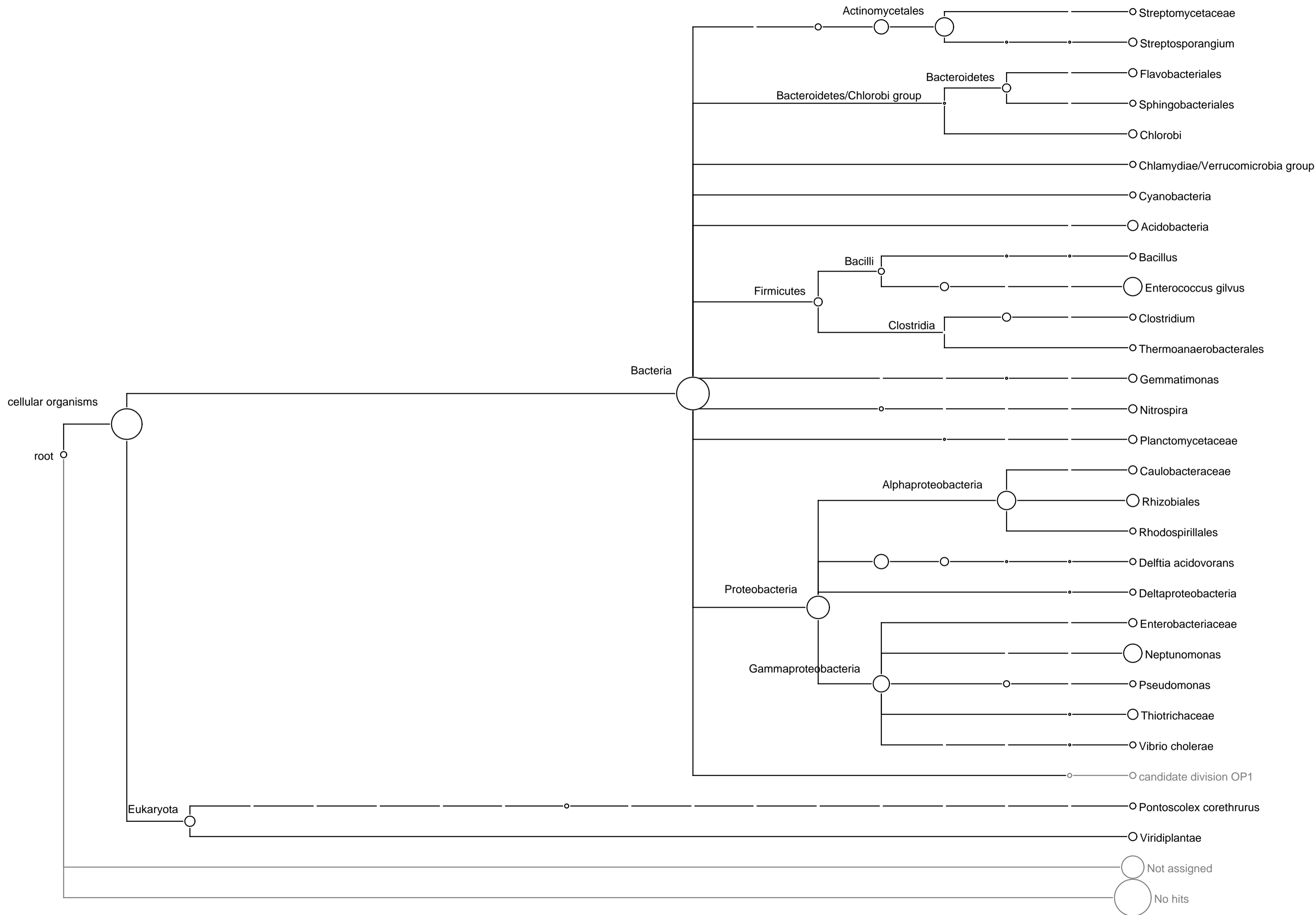

Supplement: Additional file 4 — MEGAN analysis of the reads mapped to the Yersinia pestis genome. The reads are non-specific for the pathogen. [file 1756-0500-7-111-S4.pdf]

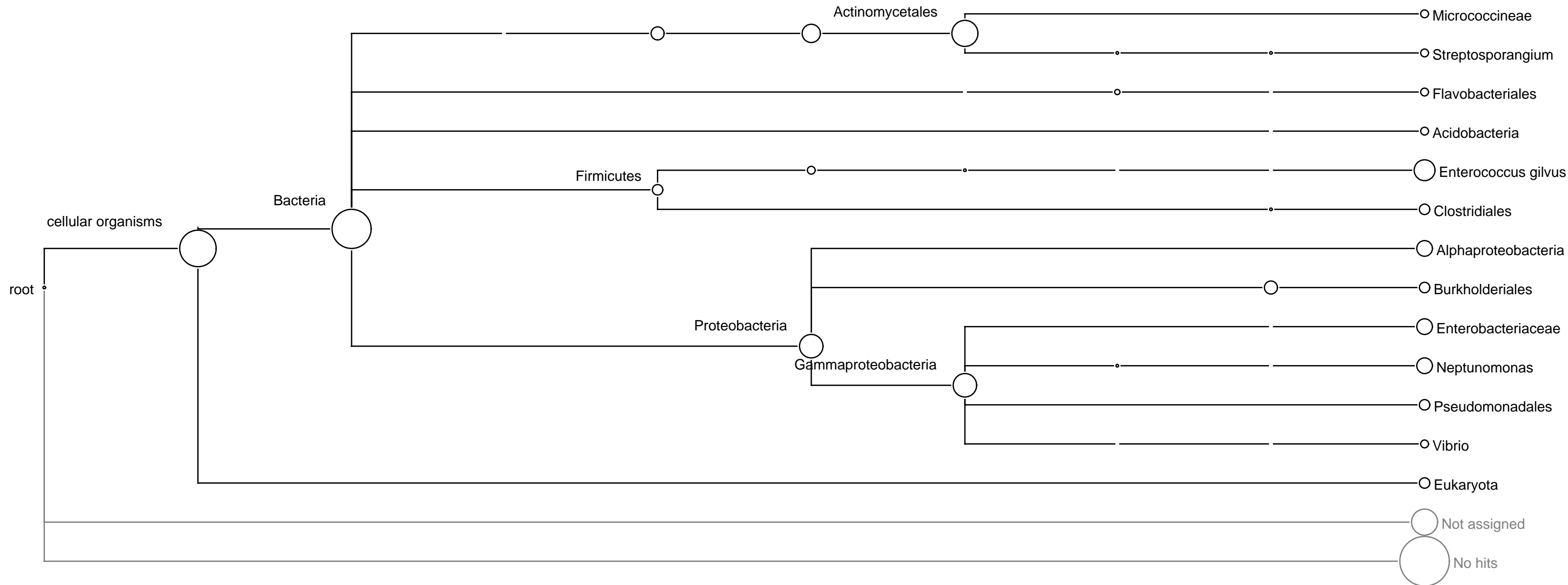

Supplement: Additional file 5 — MEGAN analysis of the reads mapped to Rickettsia genomes. The reads are non-specific for the pathogens. Instead, they correspond to common environmental organisms. [file 1756-0500-7-111-S5.pdf]

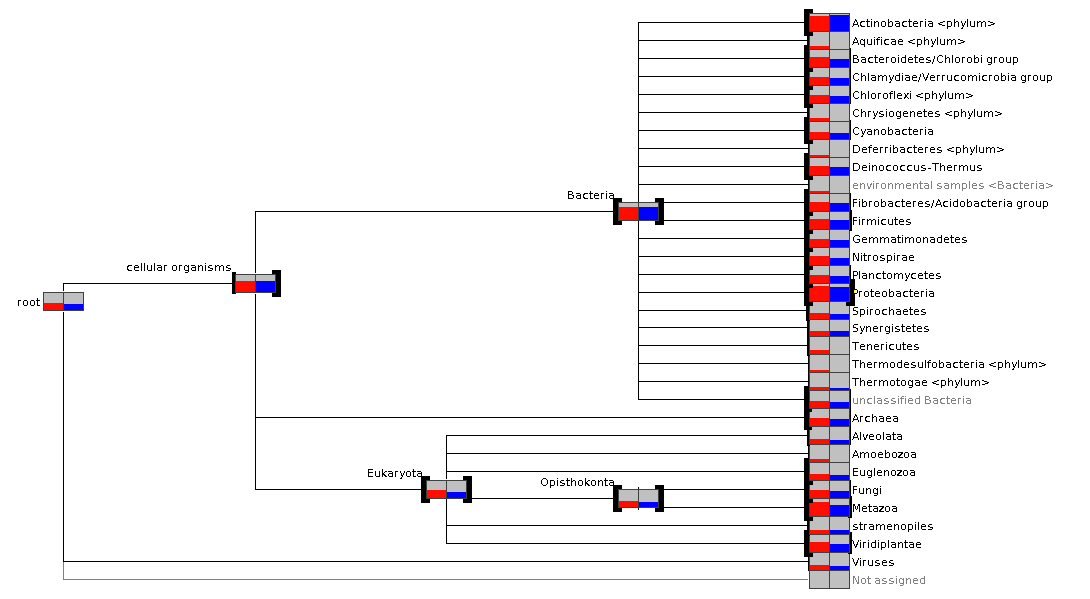

Supplement: Additional file 6 — MEGAN comparison of the BLAST hits between the Grand Plaza (blue) and Churchyard (red) populations. Significant differences in relative species prevalence are highlighted in black. The vast majority of the DNA sequences are derived from the environment, obscuring any ancient pathogen signal. [file 1756-0500-7-111-S6.png]
